# Ordered SnO_2_ nanotube arrays of tuneable geometry as a lithium ion battery material with high longevity†

**DOI:** 10.1039/c9na00799g

**Published:** 2020-02-13

**Authors:** Ying Zhuo, Sarah Tymek, Hong Sun, Maïssa K. S. Barr, Lionel Santinacci, Julien Bachmann

**Affiliations:** Friedrich-Alexander University of Erlangen-Nürnberg, Department of Chemistry and Pharmacy, Chemistry of Thin Film Materials, IZNF Cauerstr. 3 91058 Erlangen Germany julien.bachmann@fau.de; Aix Marseille Univ., CNRS, CINaM Marseille France; Saint Petersburg State University, Institute of Chemistry Universitetskii pr. 26 198504 Saint Petersburg Russian Federation

## Abstract

Ordered arrays of straight, parallel SnO_2_ nanotubes are prepared by atomic layer deposition (ALD) on inert ‘anodic’ aluminum oxide porous membranes serving as templates. Various thicknesses of the SnO_2_ tube walls and various tube lengths are characterized in terms of morphology by scanning electron microscopy (SEM), chemical identity by X-ray photoelectron spectroscopy (XPS) and phase composition by X-ray diffraction (XRD). Their performance as negative electrode (‘anode’) materials for lithium-ion batteries (LIBs) is quantified at different charge and discharge rates in the absence of additives. We find distinct trends and optima for the dependence of initial capacity and long-term stability on the geometric parameters of the nanotube materials. A sample featuring SnO_2_ tubes of 30 µm length and 10 nm wall thickness achieves after 780 cycles a coulombic efficiency of >99% and a specific capacity of 671 mA h g^−1^. This value represents 92% of the first-cycle capacity and 86% of the theoretical value.

## Abstract 2

Ordered arrays of straight, parallel SnO_2_ nanotubes are prepared by atomic layer deposition (ALD) on inert ‘anodic’ alumina porous membranes serving as templates. The tunable geometry yields exceptional capacity retention after hundreds of cycles.

## Graphical abstract

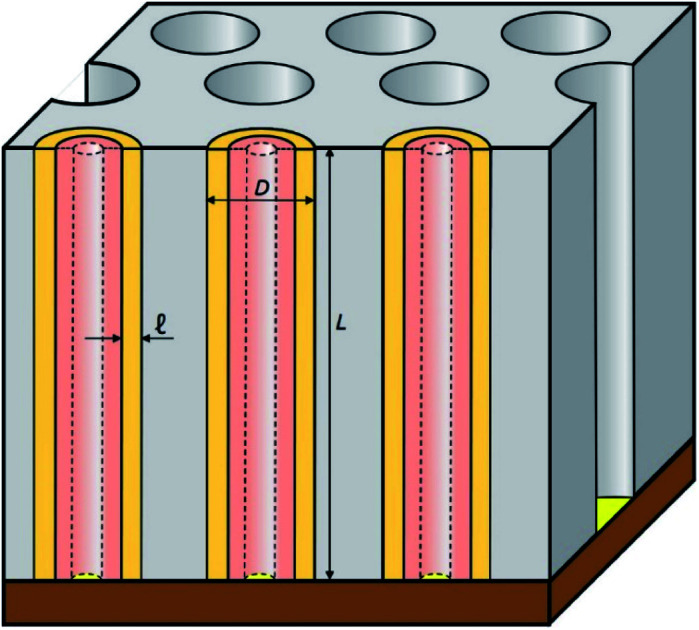

## Introduction

Owing to environmental concerns (global warming and pollution) associated with the exploitation of fossil fuels, renewable energy sources such as wind and solar energy have provided an increasing contribution to the energy mix.^1^ Their inherently intermittent nature and unequal geographic availability render the storage of energy crucial.^2^ Water electrolysis^3–5^ and batteries^6–8^ represent adequate storage technologies. In particular, rechargeable lithium-ion batteries (LIBs)^9–11^ are attractive for various applications,^12,13^ especially mobile and portable devices. Increases in the specific capacity of commercial LIBs have been mostly based on the positive electrode (‘cathode’), whereas the negative counterpart has remained carbon-based in the past several years. Thus, replacing carbonaceous material with alternatives offering a significantly improved specific capacity would represent a step change in electrochemical energy storage.^12,14^

Among the promising alternatives for the negative electrode, silicon and SnO_2_ feature prominently. Both materials actually base on the same set of reversible lithiation/delithiation events, described (for Sn) in eqn (1):^9,15–17^

1 Sn + *x*Li^+^ + *x*e^−^ → Li_*x*_Sn, (*x* ≤ 4.4)

Often, however, the starting material used in the preparation of the electrode is not metallic Sn but the decently conductive oxide SnO_2_, instead. Therefore, the following irreversible reduction must take place in the first cycle (or over the first few cycles), eqn (2):^9,15–17^

2 SnO_2_ + 4Li^+^ + 4e^−^ → Sn + 2Li_2_O

Considering the faradaic current associated with the reversible lithiation, eqn (1), and reporting it to the mass of SnO_2_ originally present yields a value of 782 mA h g^−1^ for the theoretical specific capacity often cited:^7,18,19^3
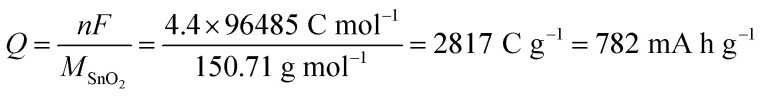


This value dwarfs graphite (372 mA h g^−1^) despite the fact that the mass of oxide is considered (not only the pure element).^7,18,19^ Despite the advantageous capacity number, Sn-based (and Si-based) electrodes have been hampered by their lack of stability so far.^12,20^ Indeed, they suffer from the large volume variation of ∼300% that occurs upon lithiation and delithiation.^12,21,22^ The strong internal stresses caused by it within the electrode material fracture the solid particles and cause the disintegration of the electrode upon repeated charge and discharge. This is associated with drastic capacity losses over tens or hundreds of cycles. The most promising recent strategies to address this issue have been the use of finely divided particulate SnO_2_ (in order to allow for volume expansion and shrinkage)^23,24^ mostly in combination with complementary solids providing electrical conduction in a composite,^16^ for example in SnO_2_-carbonaceous hybrids,^17,25–28^ or mixed with other metal oxides or metals.^29–34^Table 1 summarizes the state of the art for SnO_2_-based electrode materials. This comparison shows that the current best systems exhibit significant capacity losses unless the total number of charge–discharge cycles performed is limited and/or the rates of charge and discharge are reduced significantly. The various types of nanostructuring explored so far have not been sufficiently well controlled in order to allow for the significant volume changes without deleterious pulverization of the solid.

**Table 1:** Summary of the performance and longevity of nanostructured SnO_2_ or SnO_2_ composites as negative electrode materials for lithium ion batteries

Structure	Current	Cycle number *N*	Cycling voltage *vs.* Li/Li^+^/V	Capacity retention	Reference
3 nm SnO_2_ nanoparticles	1732.5 mA g^−1^ for charging, 60 mA g^−1^ for discharging	60	0–1.2	Almost 100%	10
4 nm SnO_2_ nanoparticles				73%	
8 nm SnO_2_ nanoparticles				3%	
SnO_2–*x*_ nanoparticles	200 mA g^−1^	100	0.01–3.0	44%	16
SnO_2–*x*_ nanoparticles + rGO				71%	
SnO_2_ nanowires	100 mA g^−1^	50	0.05–1.5	26%	23
SnO_2_ nanowires	400 mA g^−1^	100	0.005–1.5	<1%	24
SnO_2_ in TiO_2_ (wires in tubes)		500		56%	
		1000		45%	
SnO_2_ + Li_2_O nanoparticle mixture	298.8 mA g^−1^	200	0.01–3.0	70%	35
SnO_2_ nanoparticles	0.05 mA cm^−2^	80	0.005–2.0	10%	36
SnO_2_ nanotubes				56%	
Short SnO_2_ nanotubes	100 mA g^−1^	30	0.005–1.5	25%	37
Long SnO_2_ nanotubes				20%	
Sn nanocluster-covered SnO_2_ nanowires	100 mA g^−1^	40	0.0–2.2	70%	38
SnO_2_ nanosheets hollow spheres	160 mA g^−1^	50	0.01–1.2	66%	39
SnO_2_ nanoboxes	156 mA g^−1^	40	0.01–2.0	73%	40
10 nm ALD-SnO_2_ on TiC sheets	500 mA g^−1^	50	0.01–3.0	35%	41
SnO_2_ nanorods	78.1 mA g^−1^	100	0.005–2.5	74%	42
ALD-deposited SnO_2_ layer	0.1 mA cm^−2^	500	0.005–0.8	93%	43
			0.005–1.5	11%	
			0.005–2.5	27%	
SnO_2_ nanoparticles + rGO	150 mA g^−1^	100	0.01–3.00	31%	44
SnO_2_ nanoparticles + rGO + HfO_2_ 6 ALD cycles				43%	
SnO_2_ hollow sphere (HS)	100 mA g^−1^	60	0.005–3.0	12%	45
SnO_2_ HS with GO wrapping				48%	
SnO_2_ HS with Al_2_O_3_ coating				31%	
SnO_2_ HS + GO wrapping + Al_2_O_3_ coating				53%	
4 to 6 nm SnO_2_ nanoparticles	100 mA g^−1^	50	0.005–1.0	92%	46
**SnO**_**2**_ **nanotubes**	**391 mA g** ^**−1**^	**780**	**0.02–2.8**	**92%**	**This work**

**Fig. 1:**
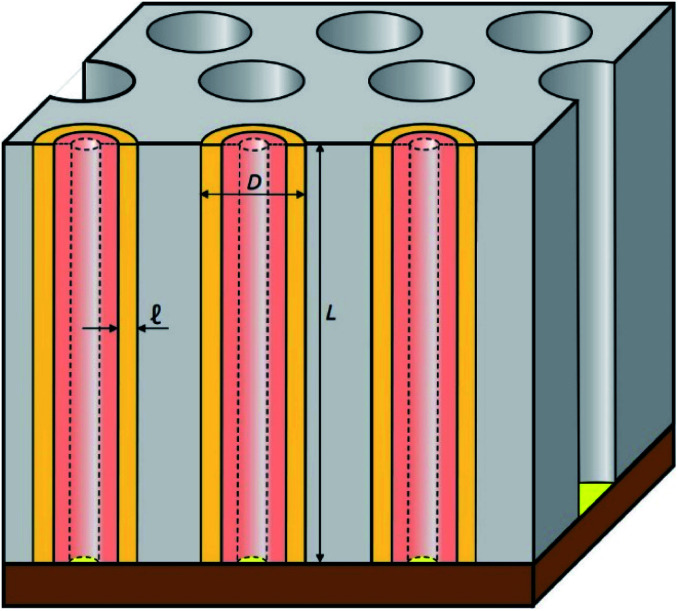
Schematic presentation of the nanostructured SnO_2_ electrodes studied in this work. ALD-SnO_2_ nanotubes (pale orange) are embedded within an anodic aluminum oxide (AAO) matrix (gray). The void space in the center of each nanotube allows for the volume expansion of the electroactive material upon lithiation (salmon color). Gold is sputtered as a thin electrical contact (yellow). The sample is then glued on Cu foil (brown). Every geometric parameter of this ordered structure is independently tunable: outer tube diameter *D*, wall thickness ℓ, length *L*.

In this work, we introduce arrays of parallel SnO_2_ nanotubes (Fig. 1), fabricated by atomic layer deposition (ALD) in ‘anodic’ aluminum oxide (AAO) membranes as inert templates. Anodization not only yields a high quality of cylindrical, straight and ordered pores, it also allows the experimentalist to vary the pore geometry accurately: the pore diameter depends on anodization voltage and electrolyte, and the pore length on the anodization duration.^47^ ALD provides the ideally complementary techniques as it generates homogeneous coatings in three-dimensional structures, including highly porous ones, and enables one to adjust the coating thickness with sub-nanometer accuracy.^48–51^ This design presents several advantageous features for a systematic study of how geometry affects performance and stability. Firstly, the straight geometry of the tubes defines a continuous electron transport path from the electrical contact to any point of the electroactive solid, thus rendering additives such as graphite (mostly used in other studies) unnecessary. This simplifies the analysis and interpretation of the results significantly. Secondly, the void internal volume of our SnO_2_ nanotubes serves to take up the volume expansion occurring during lithiation, thus allowing the material to better endure the volume variations caused by charge and discharge. Thirdly, the transport distances for lithium from the electrolyte interface to any point of the solid are short and controllable by the tube wall thickness. We will show in the following that this geometry yields significant improvements in stability over systems published so far (see Table 1).

## Experimental section

### Materials

Ethanol, perchloric acid (70%), phosphoric acid, chromium(vi) oxide, copper(ii) chloride dihydrate, hydrochloric acid (37%), nitric acid (65%), hydrogen peroxide, tetrakis(diethylamido)tin(iv), metallic Li, lithium hexafluorophosphate (LiPF_6_) solution and Cu foil were purchased from abcr, VWR or Sigma Aldrich and used as received. Aluminum plates (0.5 mm thick) were provided by Smart Membranes. Water was purified in a Millipore Direct-Q System. Au target for sputtering was supplied by Stanford Advanced Materials. Silicon wafers coated with an oxide layer were from Silicon Materials Inc. CR2032 cases, stainless steel spacers and springs were purchased from MTI Corporation. Celgard 2325 separators were from EL-Cell GmbH.

### Preparation of anodic aluminum oxide (AAO) membranes

AAO membranes used as the template for growing SnO_2_ nanotubes were fabricated by two-step anodization.^29^ First of all, aluminum plates (2 × 2 cm^2^) were electropolished in a 1 : 3 (v/v) mixture solution of concentrated HClO_4_ and ethanol while stirring at 20 V for about 5 min. Afterwards, these aluminum plates were anodized in 0.5 wt% H_3_PO_4_ solution for 1 h at +195 V at 0 °C, after which anodization was continued in 1 wt% H_3_PO_4_ for another 23 h. The anodization was performed in a two-electrode system, in which the aluminum plates were sandwiched between a PVC beaker with O-ring on top and a Cu plate underneath, used as the electrical contact. The PVC beaker contained the H_3_PO_4_ solution and was covered by a cap providing the counter-electrode (silver wire mesh) and a mechanical stirrer. After this first anodization, chromic acid solution (0.18 M CrO_3_ in 6 wt% H_3_PO_4_) was added to the beakers to remove the irregularly grown aluminum oxide nanopores for 24 h at 45 °C. The second anodization was carried out at +195 V and at 0 °C, first for 1 h in 0.5 wt% H_3_PO_4_ and subsequently for an adjustable duration in 1 wt% H_3_PO_4_. The duration of this second anodization determined the length of the AAO matrix pores. The aluminum substrate was later removed with 0.7 M CuCl_2_ in 10% HCl. 10 wt% H_3_PO_4_ solution was used to remove the barrier layer of the AAO membrane at 45 °C (35 min from the back side) and perform pore widening (full immersion in H_3_PO_4_ for another 35 min). Finally, laser-cut circles of AAO templates were dried overnight at 45 °C.

### Deposition of thin films

Deposition of the thin SnO_2_ layer along the inner walls of the AAO template pores to build up SnO_2_ nanotubes was performed by ALD in a commercial Gemstar-6 XT ALD reactor from Arradiance at 200 °C with tetrakis(dimethylamino)tin(iv) (TDMASn) and hydrogen peroxide (30% aqueous solution) as precursors. TDMASn and H_2_O_2_ were maintained in stainless steel bottles at 60 °C and at room temperature, respectively. Subsequently, a thin gold contact layer (approximately 50 nm) was sputtered onto one side of the sample in a torr CRC 622 sputter coater. The loading of SnO_2_ was determined as a difference by mass measurements of the sample before and after ALD with an analytical balance (Sartorius micro, nominal accuracy ±0.001 mg, observed reproducibility ±0.003 mg). The accuracy of the mass determination of loading is evaluated to be ±0.005 mg. For example, the sample with *L* = 30 µm, ℓ = 10 nm has 0.650 mg of SnO_2_ in 4.062 mg of AAO membrane.

### Characterization

The thicknesses of SnO_2_ layers were determined on Si(100) wafers by spectroscopic ellipsometry with a SENPro from SENTECH. Scanning electron microscopy (SEM) and energy-dispersive (EDX) spectroscopy were implemented with an Jeol JSM 6400 upgraded with a LaB_6_ cathode and SDD X-ray detector. The crystal structure of the samples was investigated by X-ray diffraction (XRD) with Cu Kα_1_ radiation (*λ* = 1.54056 Å) on a Bruker D8 Advance diffractometer equipped with a LynxEye XE-T detector. The measurements were performed in the regular Bragg–Brentano geometry for porous samples and in grazing incidence (0.6°) for planar ones. X-ray photoelectron spectroscopy (XPS) was carried out with monochromatized Al Kα X-ray photoelectron spectroscopy (PHI Quantera II, Japan).

### Electrochemical studies

The nanostructured SnO_2_ with AAO template were laser-cut with a GCC LaserPro Spirit LS Laser from their Al frame and into smaller pieces. The samples were then glued onto Cu foil by using double-sided adhesive and conducting Cu tape. The area of the sample was defined by a circular window laser-cut into chemically resistant and electrically insulating polyamide tape (Kapton®). They were complemented with a Li foil and 1.0 M LiPF_6_ in 50 : 50 (v/v) ethylene carbonate and diethyl carbonate, which was purchased from Sigma Aldrich, in CR2032 coin cells (from MTI Corporation) for galvanostatic charge/discharge curves at different current rate in a range of 0.02 V to 2.8 V. Voltammetry and impedance spectroscopy datasets were recorded with an additional lithium metal reference electrode in a three-electrode configuration in a cell from EL-cell. Cyclic voltammograms were performed at a scan rate of 0.1 mV s^−1^ in the potential range 0.02 V to 2.8 V, while galvanostatic electrochemical impedance spectroscopy measurements were completed with an amplitude of 0.99 µA over the frequency range of 0.1 Hz to 1 MHz after different cycling numbers.

## Results and discussion

**Fig. 2:**
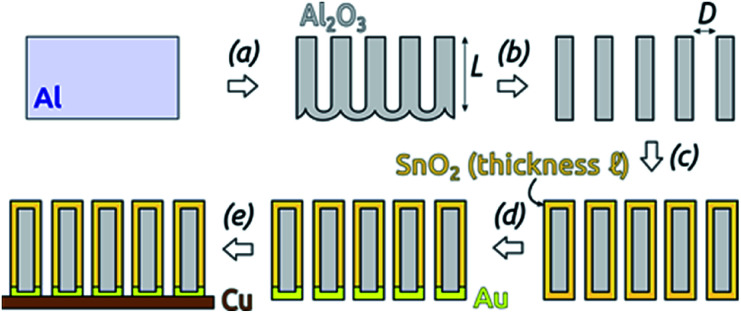
Preparation of SnO_2_ nanotubes: (a) two-step anodization of Al foil (defining the tube length *L*) and subsequent wet chemical removal of metallic Al. The length of the AAO membranes was defined by this step. (b) Wet chemical Al_2_O_3_ barrier layer removal and pore widening (defining the outer tube diameter *D*). (c) ALD of SnO_2_ (defining the tube wall thickness ℓ). (d) Sputter-coating of Au. (e) Laser-cutting of the sample and gluing with conductive Cu adhesive tape.

The preparative procedure towards ordered arrays of straight, cylindrical SnO_2_ nanotubes by atomic layer deposition (ALD, 50 to 250 cycles corresponding to 10 nm to 50 nm)^48,49,52^ in anodic aluminum oxide (AAO) membranes as templates is presented in Fig. 2. In short, the outstanding ability of ALD to coat deep pores (for the electroactive material) is complemented by the property of physical deposition methods (sputtering) of only coating one sample face (for the electrical contact). For this study, we will consider of SnO_2_ nanotube lengths of 30 µm to 150 µm obtained by anodizing for 10 h to 50 h. We maintain a constant tube outer diameter (*D*) of 390(±10) nm for a center-to-center distance of 450 nm.

**Fig. 3:**
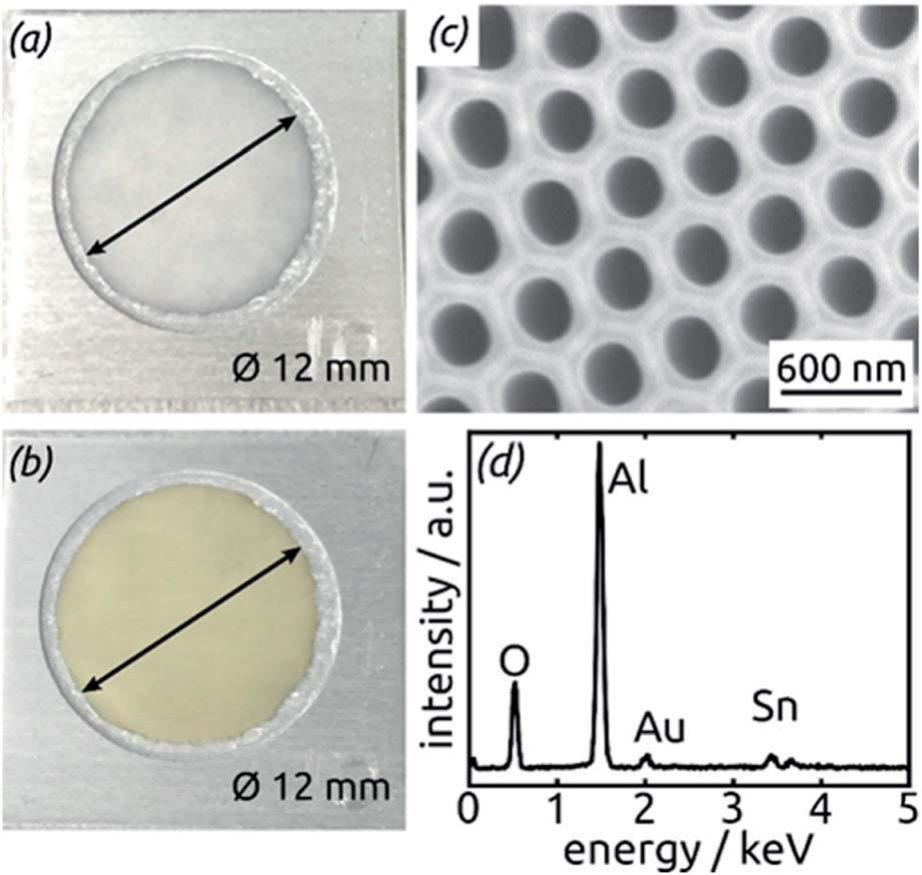
(a) Macroscopic photograph of a transparent, bare AAO membrane before ALD. (b) Same sample after SnO_2_ ALD. (c) SEM micrograph of a sample in top view after all preparation steps of Fig. 2. (d) EDX spectrum exhibiting the presence of Sn.

Fig. 3a and b compares the macroscopic appearance of an AAO membrane before and after SnO_2_ ALD coating (10 nm as determined by spectroscopic ellipsometry on a planar reference sample). The slightly yellow color of the semiconductor is evident and homogeneous across the full sample diameter (12 mm). Of course, thicker coatings result in darker shades of yellow. The high structural quality of the samples on the microscopic scale is presented on Fig. 3c, where the regularly ordered AAO pores, coated with SnO_2_, are evident in top view. The presence of the SnO_2_ layer is proven by EDX spectroscopy (Fig. 3d).

**Fig. 4:**
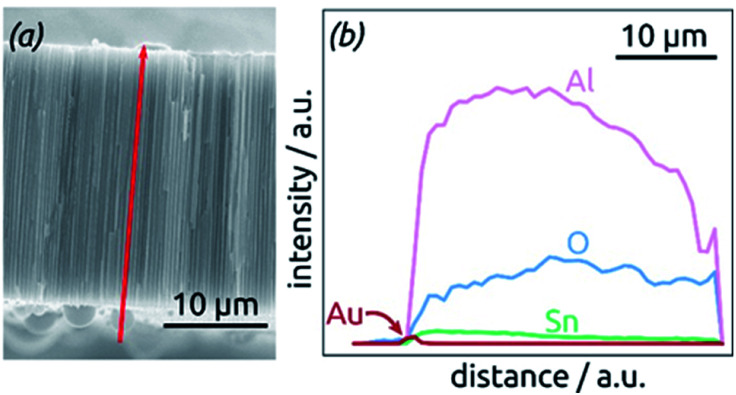
Element analysis of a sample by EDX spectroscopy. (a) SEM micrograph of the cross-section analyzed, displaying the profile analyzed as a red arrow. (b) EDX profile for the elements Al, O, Sn and Au along the path defined in (a).

The homogeneity of SnO_2_ ALD coating along the pore length is proven by an EDX profile recorded along the cross-section of the SnO_2_ sample, Fig. 4. The Sn signal is continuous from one side of the sample to the other, albeit not perfectly constant. It stands in stark contrast, however, to the Au signal associated with the electrical contact, which is nicely located on one sample surface only.

**Fig. 5:**
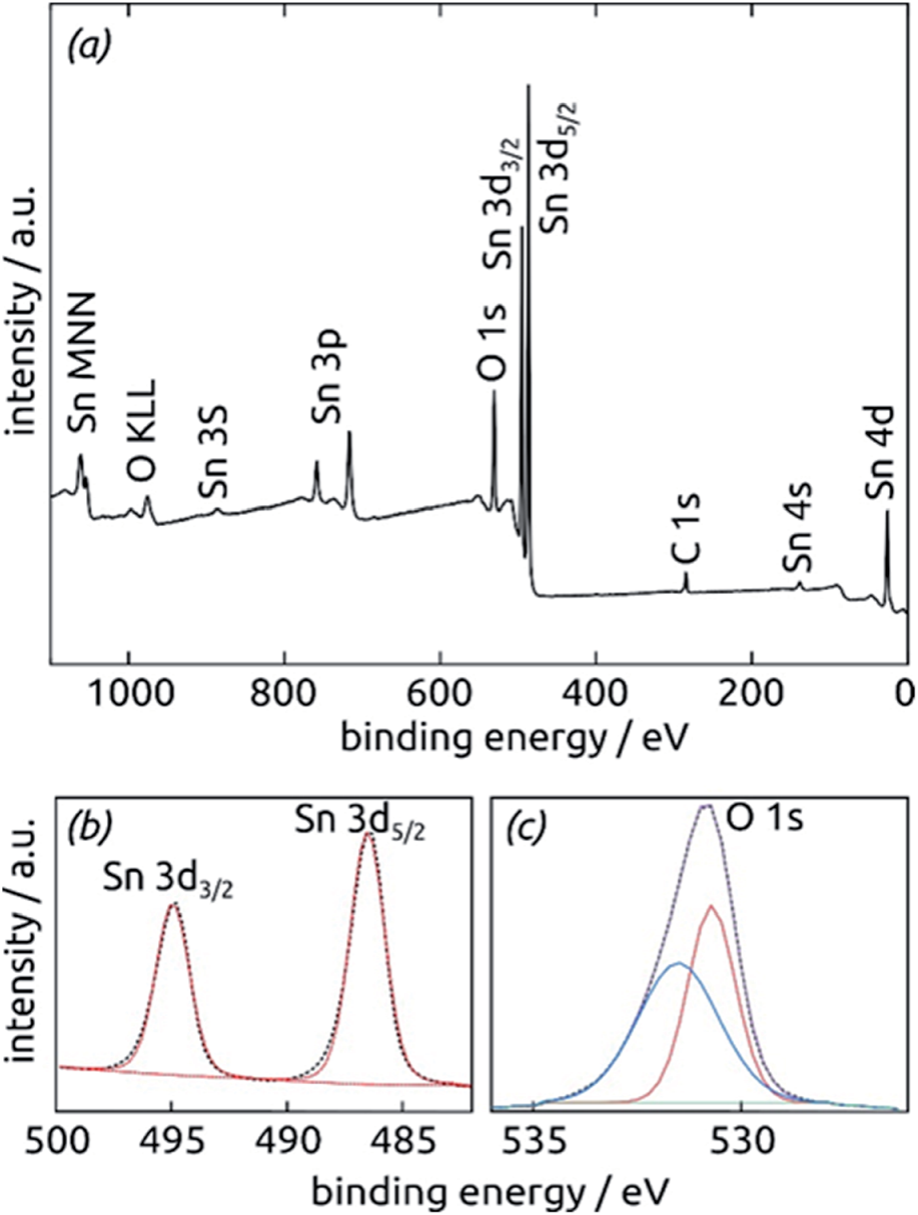
X-ray photoelectron spectra of a SnO_2_ film (10 nm) on a clean Si (100) surface. (a) XPS survey spectrum indicating the presence of all expected elements. (b) XPS Sn 3d region fitted by one single spin-split doublet. (c) XPS O 1s region fitted by two peaks.

Further insight into the chemical identity of our ALD-deposited material is provided by X-ray photoelectron spectroscopy (XPS, Fig. 5). The survey spectrum (Fig. 5a) exhibits the elements Sn and O as expected, in addition to the weak C 1s peak always present at 284.8 eV for samples handled in air. No other impurities are observed. The ratio between Sn and O is found to be 1 : 2.7, larger than the value of 2 expected for SnO_2_. The XPS Sn 3d line (Fig. 5b), however, is a perfect spin-split doublet at 486.52 eV and 494.94 eV, the position expected of SnO_2_.^53,54^

Thus, tin centers are homogeneously in oxidation state +IV and bonded to oxygens. The O 1s region, in contrast to this, must be fitted with two distinct species. The peak at 530.70 eV perfectly corresponds to perfectly stoichiometric SnO_2_. The broader peak at higher energies falls into the range expected of –(OH) groups.^55,56^ These observations are consistent with a partial hydration of SnO_2_ in the surface-near region.

**Fig. 6:**
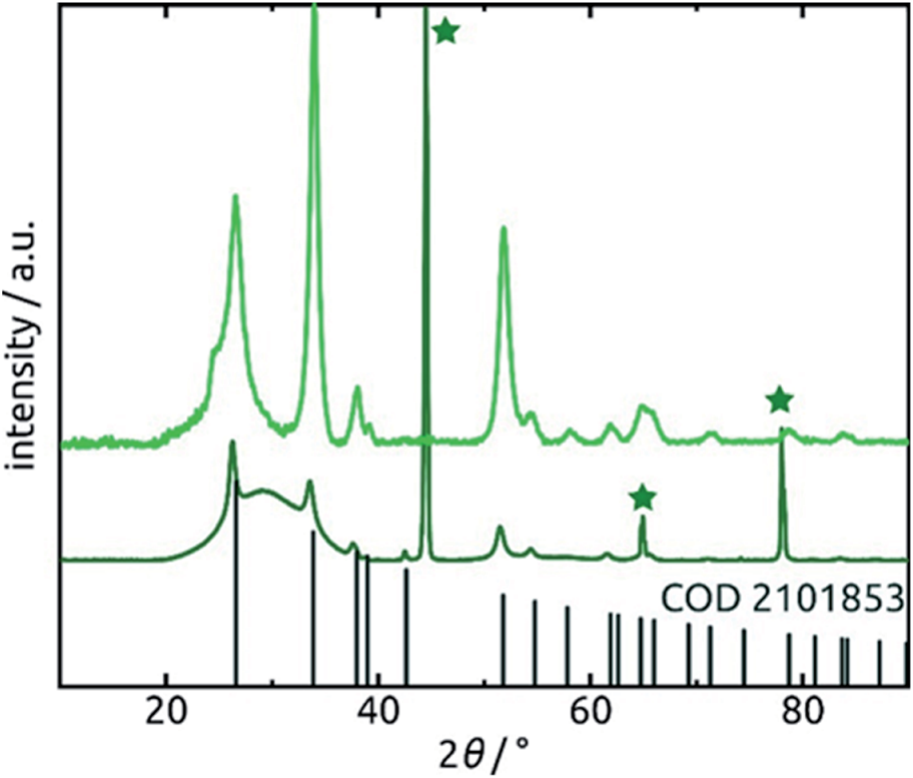
X-ray diffraction patterns of 30 nm of SnO_2_ deposited on an AAO substrate and a planar Si wafer. Light green: grazing-incidence XRD (GIXRD) of the planar film recorded under 0.6° incident angle. Dark green: regular (Bragg–Brentano) XRD of the nanotube sample. The peaks associated with metallic aluminum are marked by a star. The positions of peaks expected of SnO_2_ (COD 2101853) are marked.

Further confirmation of the identity of the ALD material is provided by X-ray diffraction (XRD). Fig. 6 reveals that crystalline SnO_2_ is obtained from ALD without annealing necessary, as has been reported before for layers of sufficient thickness and depending on deposition temperature and substrate.^57,58^ Therefore, the amount of hydrated material (presumably amorphous) is low in the bulk. The very broad signal stretching from 20° to 40° and evident of a large amount of amorphous material can be attributed to the alumina matrix, since it is absent of data recorded for planar films on silicon wafers in grazing incidence (0.6°). The three sharp peaks marked by stars at 45°, 65° and 78° in the porous sample diffractogram (dark green in Fig. 6) are due to the Al frame of the AAO membrane.

**Fig. 7:**
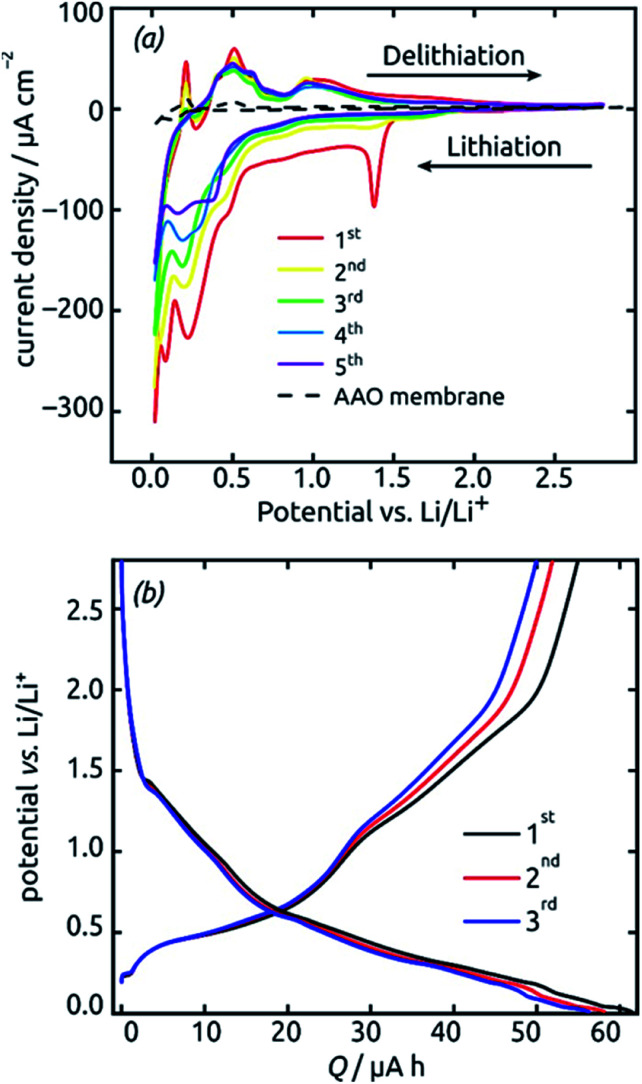
Initial electrochemical properties (three cycles) of a SnO_2_ nanotube electrode (ℓ = 10 nm, *L* = 30 µm, 4 mm macroscopic sample diameter). (a) Cyclic voltammograms recorded at a scan rate of 0.1 mV s^−1^ in the potential range 0.02 V to 2.8 V. (b) Voltage profiles recorded under a constant current of 19.8 µA (corresponding to a rate of 0.5C).

These SnO_2_ nanotubes can be exploited as lithium ion battery materials after addition of a gold electrical contact, without the need to remove them out of the matrix or to complement them with any electron and/or ion conducting additives as is usually performed. The conditions for it are a high electrical conductivity^57,59^ and a continuous, straight transport pathway along the tube axis. Fig. 7 demonstrates that indeed, such a sample is electrochemically active as is. Starting from +2.8 V *vs.* a lithium foil in LiPF_6_ electrolyte, the first cyclic voltammogram exhibits the irreversible reduction peak of SnO_2_ to metallic Sn at 1.38 V (eqn (2) in the Introduction), which is very characteristic and well established in the field^9,16,17^ The complete disappearance of this reaction over the subsequent two cycles is indicative of an outstanding accessibility of all SnO_2_ present to both electrons and lithium ions. An additional irreversible feature that disappears over the first few cycles is the shoulder observed at 0.48 V. This feature is also precedented in the SnO_2_ literature and has been attributed to the formation of the solid–electrolyte interface (SEI) *via* electrolyte decomposition.^17^

The peaks attributed to the reversible lithiation of Sn (eqn (1) in the Introduction) are observed at 0.22 V and 0.08 V during the first cathodic sweep, and they shift somewhat to more positive potentials over the first five cycles.^9,16^ Both the potential values and this slight shift are consistent with SnO_2_-based electrodes studied so far.^9,16^ The corresponding anodic features are found at approximately 0.40 V, 0.50 V, 0.60 V and 0.90 V for delithiation.^9,16^ We note that the aluminum oxide matrix is inert electrochemically, as demonstrated by a reference measurement performed in the absence of SnO_2_ (dashed black line on Fig. 7), at least after completion of the first few cycles.

The voltage profiles obtained from galvanostatic measurements performed in the same potential range display stages of low slope at the peak positions in the voltammograms, as expected. The fact that perfectly horizontal plateaus are replaced by low-slope sections is due to the rather fast charge/discharge rate (one full charge within 2 hours or 0.5C) used for the galvanostatic measurements. The galvanostatic curves allow us to quantify the degree of irreversibility caused be SEI layer formation at the early stages (over the first three cycles) to 8%, yielding (equivalently) a coulombic efficiency of 92%. The first-cycle absolute capacity of 0.235C translates to an areal capacity of 1870 mC cm^−2^ or 0.519 mA h cm^−2^ and to a gravimetric specific value of 1284 mA h g^−1^. This value is larger than the theoretical reversible capacity of 782 mA h g^−1^ on the basis of SnO_2_ (as defined in the Introduction) and is in line with the observation that in the first few cycles a significant amount of irreversible processes take place. The development of these values over time, that is, over large numbers of charge and discharge, will be described later as it depends on charge rates and geometry.

**Fig. 8:**
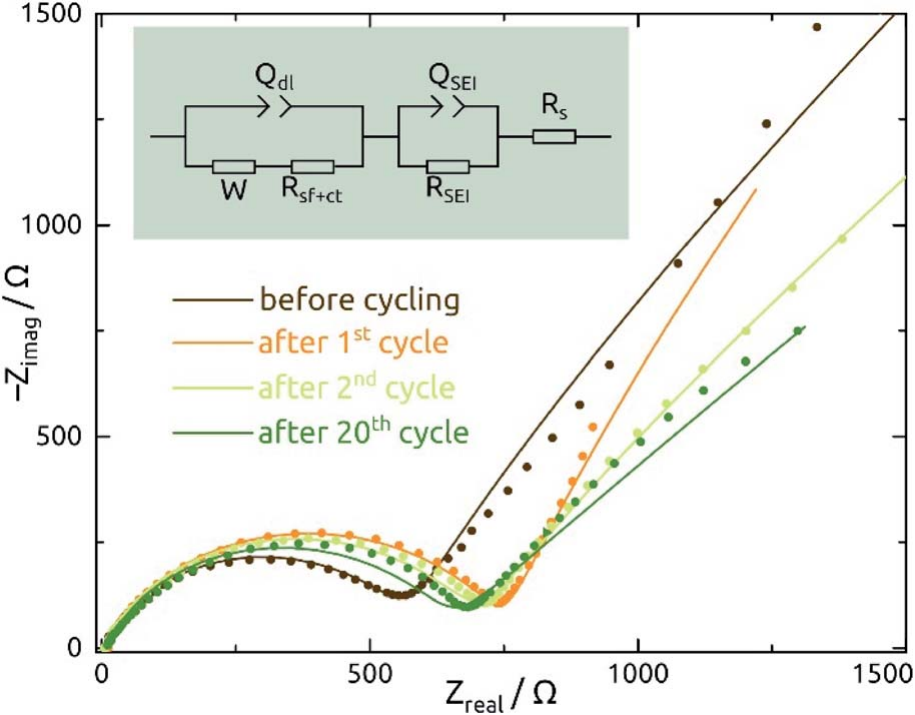
Nyquist plots of galvanostatic electrochemical impedance spectroscopy (EIS) measurements recorded of SnO_2_ nanotube electrodes (ℓ = 10 nm, *L* = 30 µm, 4 mm macroscopic sample diameter) in their delithiated state after 0, 1, 2, and 20 charge/discharge cycles (spectra are plotted in dots). The equivalent electrical circuit model shown in figure is used to fit the data, whereas the *Q*_SEI_/*R*_SEI_ element is abandoned for the dataset recorded before cycling. *W*: Warburg diffusion; *Q*_dl_: constant-phase element due to double layer; *R*_ct_: charge transfer resistance; *Q*_SEI_, *R*_SEI_: constant-phase element and resistance due to the solid/electrolyte interface (SEI) layer; *R*_s_: series resistance.

Galvanostatic electrochemical impedance spectroscopy measurements carried out (in a three-electrode configuration) over the frequency range of 0.1 Hz to 1 MHz after 0, 1, 2, and 20 charge–discharge cycles are presented in Fig. 8 as Nyquist plots. The data consist of a semicircle and a straight line at lower frequency range, attributed to charge transfer impedance at the electrode/electrolyte interface and transport of Li^+^, respectively.^27^ First of all, the series resistance is reasonably low (<10 Ω), indicating a good electrical contact. The curve changes most significantly from before the first cycle to after it, as expected based on the irreversible chemical changes (reduction to Sn metal and SEI layer formation) that occur during it as discussed above.

The quantitative EIS parameters presented in Table 2 derive from fits according to the equivalent electrical circuit model shown in Fig. 8 and adapted from literature precedents.^31,32,44^ We fix the *α* values of constant-phase elements to 0.8 for *Q*_SEI_ and 0.9 for *Q*_dl_. The SEI-related elements are ignored for the pristine sample, which does not exhibit any SEI yet. After the first cycle, the values of *Q*_SEI_ and *R*_SEI_ remain fairly constant, which indicates a stable SEI layer. *R*_ct_ decreases after cycling, again signifying properties that are stable (or even improving) upon repeated cycling (eqn (1)). The behavior of *Q*_dl_ seems to indicate a significant roughening upon initial cycling, followed by a slow but steady smoothening subsequently. Fig. S1† presents the EIS data recorded at various potentials and from SnO_2_ nanotubes with different wall thicknesses for completeness.

**Table 2:** Parameters fitted from EIS spectra in Fig. 8 recorded after various numbers *N* of charge–discharge cycles

*N*	*W*/µS s^0.5^	*R*_s_/Ω	*Q*_SEI_/µS s^0.8^	*R*_SEI_/Ω	*Q*_dl_/µS s^0.9^	*R*_sf+ct_/Ω
0	516 ± 6	9.3 ± 0.1	NA	NA	1.6 ± 0.1	442.0 ± 4
1	361 ± 5	7.8 ± 0.2	3.8 ± 0.06	738.0 ± 28	417.6 ± 0.1	79.4 ± 11
2	623 ± 1	6.2 ± 0.1	4.0 ± 0.04	696.2 ± 22	279.7 ± 0.1	66.2 ± 1
20	1129 ± 1	9.5 ± 0.2	3.9 ± 0.08	637.0 ± 30	211.2 ± 0.1	30.9 ± 4

**Fig. 9:**
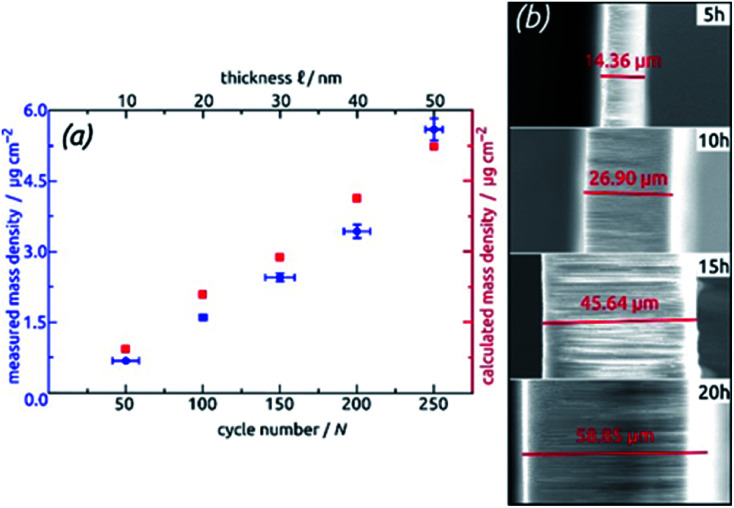
Geometric control of SnO_2_ nanotube electrodes. (a) Demonstration of varying ℓ *via* the number *N* of ALD cycles: the graph shows the SnO_2_ loading determined gravimetrically and compared to the values calculated based on geometric considerations. (b) Demonstration of varying *L via* the anodization duration: the micrographs present cross-section views of the samples.

For the systematic investigation of how electrochemical performance (specific capacity and stability) depends on geometric parameters, we need to generate samples with various geometries. Fig. 9 displays how the tube wall thickness ℓ, on the one hand, and the tube length *L*, and the other hand, are controlled experimentally. The former is adjusted *via* the number *N* of ALD cycles performed but is also accessible experimentally *via* direct gravimetric measurements. The graph (Fig. 9a) shows how the SnO_2_ loadings determined directly increase linearly with *N* and correlate with the values expected based on geometric considerations—for a given value of *L*. Independently of this, *L* can be varied for a constant ℓ as displayed by the low-magnification micrographs of Fig. 9b. The gravimetric determinations of SnO_2_ loadings are associated with an absolute uncertainty of approximately 0.005 mg, which translates to an experimental error of ±1% for the samples with the thickest coatings and ±3% for their thinnest counterparts. However, the linearity of ALD film growth can also be used to check the validity of the gravimetric determinations, as is especially useful for the thinnest (10 nm) samples. Further confirmation of the numbers has been provided by quantitative element analysis using inductively coupled plasma optical emission spectroscopy (ICP-OES) after complete dissolution of a test sample.

**Fig. 10:**
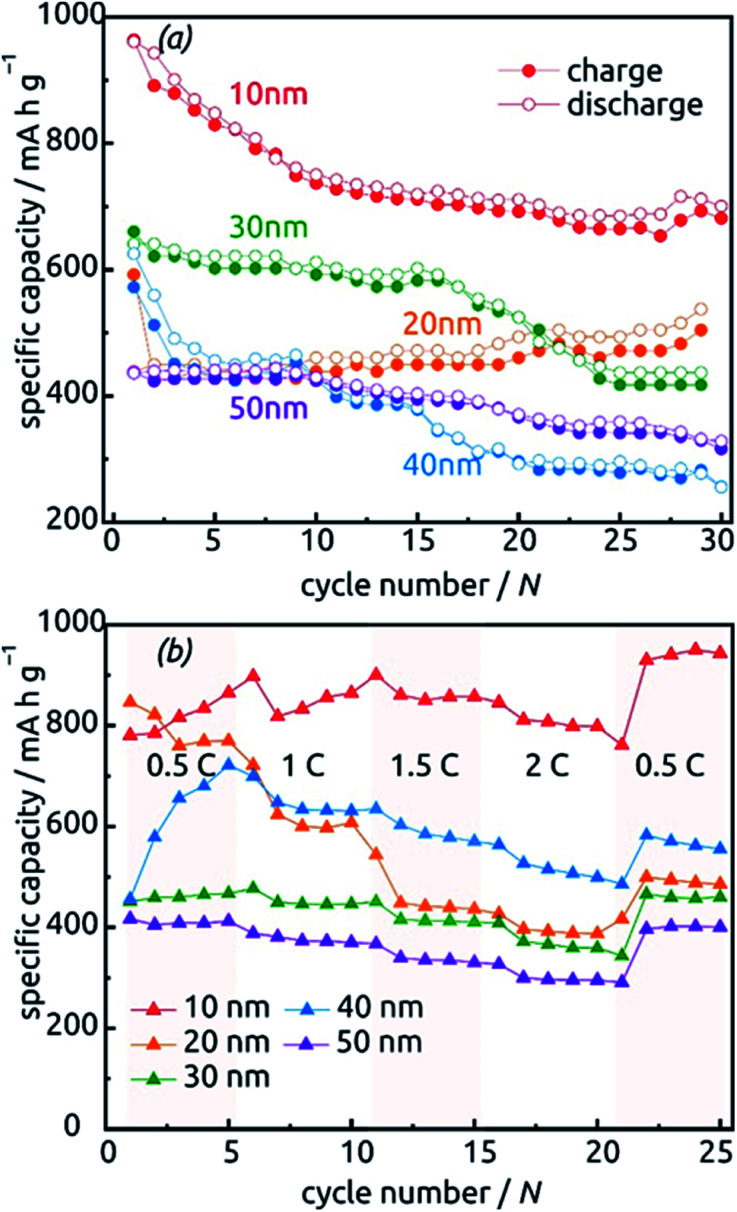
Charge–discharge behavior of SnO_2_ nanotube electrodes with various wall thicknesses between 10 nm and 50 nm. (a) Thirty cycles performed at a constant rate of 0.5C calculated based on the theoretical capacity value of 782 mA h g^−1^. (b) Performance at various C-rates.

The behavior of samples with various SnO_2_ tube wall thicknesses ℓ over the first 30 charge/discharge cycles at 0.5C is presented in Fig. 10a. Note that in this paper, we always base C-rate denominations on the theoretical specific capacity of SnO_2_ (782 mA h g^−1^). Thus, 0.5C represents 391 mA per gram of SnO_2_ as determined gravimetrically after ALD. Note also that repeating measurements on nominally identical samples allows us to evaluate the experimental uncertainty to roughly ±17% (caused to a large extent by the gravimetric determination of SnO_2_ amount), but Fig. 10 displays the data recorded on one individual batch.

All samples exhibit sub-unity coulombic efficiency and a concomitant significant capacity loss that stabilizes gradually. The most striking difference between the samples is in the gravimetric specific capacity values, with the thinnest tubes performing best. This must be due to the excellent accessibility of the full volume of electroactive material to lithium insertion. Of course, the values beyond theoretical capacity recorded for this sample over the very first cycles do not represent the reversible lithiation/delithiation of Li_*x*_Sn – they contain all additional, irreversible processes (SnO_2_ reduction to Sn, SEI layer formation, all further decomposition reactions) and are associated with a significant relative uncertainty caused by the gravimetric determination of SnO_2_ loading as described above.

The data presented in Fig. 10b, recorded on fresh samples with nominally identical geometric parameters, feature their rate stability. The loss of accessible capacity from 0.5C to 2C varies between 7.60% and 48.60%, but in most cases cycling at high rates does not permanently damage the material as it recovers its capacity in subsequent, slow cycles.

**Fig. 11:**
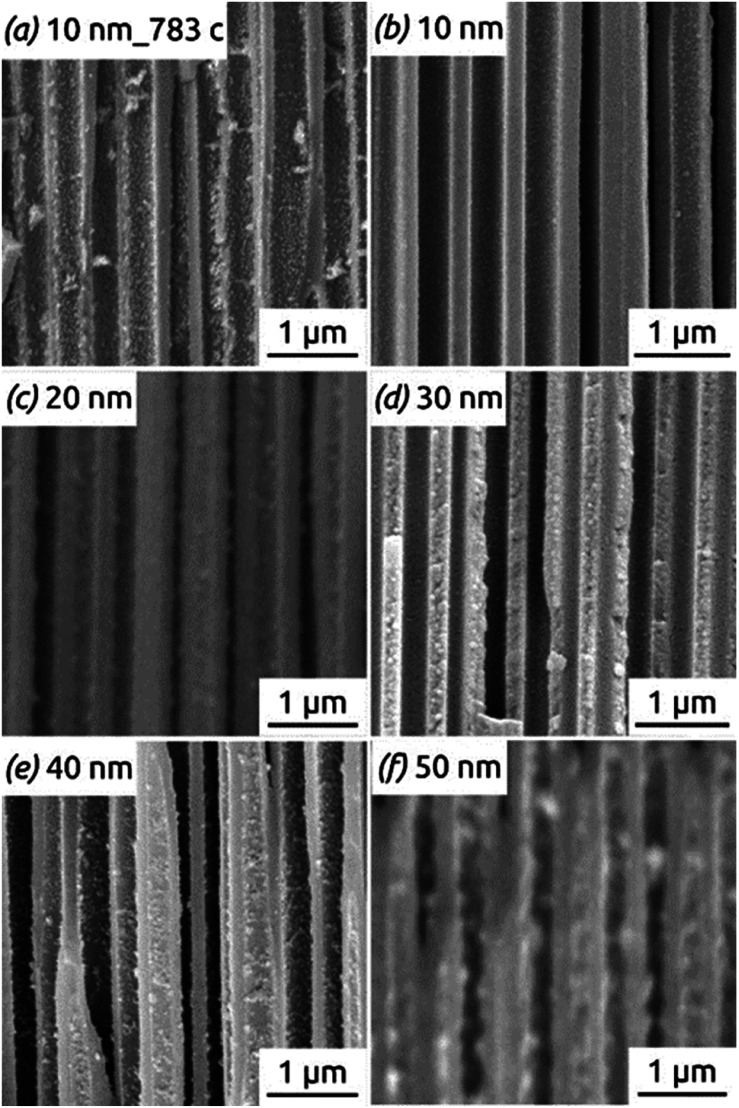
SEM images of SnO_2_ nanotube electrodes with different wall thicknesses, presented in cross-section after 100 to 783 charge/discharge cycles: (a and b) 10 nm; (c) 20 nm; (d) 30 nm; (e) 40 nm; (f) 50 nm.

Fig. 11 shows the reason for the capacity loss. A change in morphology is observed after the completion of many hundreds of cycles from perfect, hollow tubes to particles. This deterioration is particularly apparent for the tubes with thicker walls (Fig. 11f), where the large particles generated over time may even clog the pores, thus rendering some of the electroactive material inaccessible to the electrolyte. In contrast to this, thin tubes become rougher after cycling but never generate such large, separate particles (compare Fig. 11a taken after 783 cycles with Fig. 11f taken after only 100 cycles). Thus, the nanotube strategy of improving stability is most appropriate with thin walls (as expected).

**Fig. 12:**
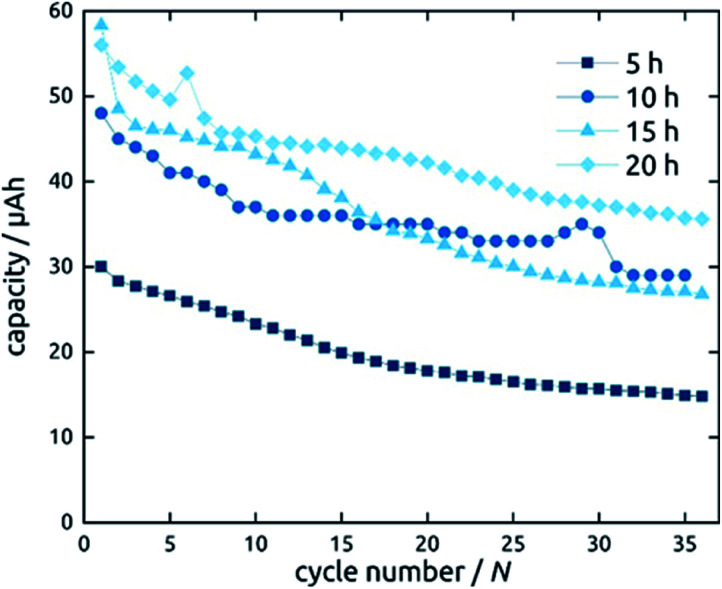
Galvanostatic charging and discharging curves of SnO_2_ nanotube electrodes with 10 nm wall thickness. The length of SnO_2_ nanotubes has been varied.

Not only the wall thickness ℓ but also the tube length *L* can be varied. Fig. 12 shows data obtained for a series of samples with various *L*: they differ in absolute capacities but converge when described in units of specific capacity (Fig. S2†), demonstrating that even with the thinnest tubes and in the absence of any conductive additive, all of the material is accessible and electroactive, as designed. Indeed, the absolute capacity increases with loading (as varied with *L* at constant ℓ) in a roughly linear manner. The behavior of the capacity loss over the initial cycles, however, is somewhat differentiated. Although each sample behaves in an individual manner, the general observation is that shorter tubes tend to reach a steady state faster. This could be due to the ohmic voltage drop present from the current collector to the top tube extremity, which spreads all processes, including the irreversible ones that have to occur in the initial cycles, over the voltage axis.

**Fig. 13:**
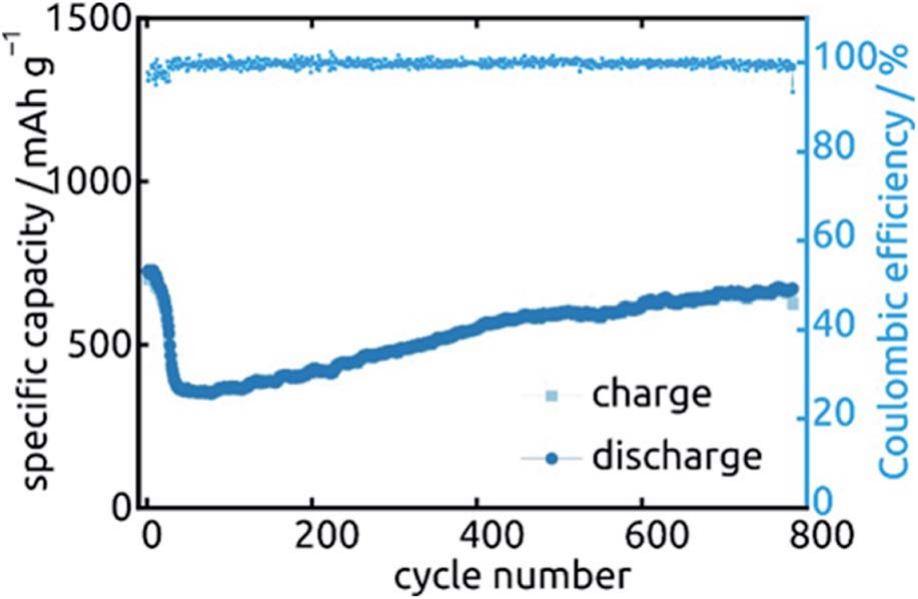
Long-term stability of a SnO_2_ nanotube sample (*L* = 30 µm, ℓ = 10 nm) during 783 cycles of galvanostatic charge and discharge.

Let us finally turn to the long-term stability of our electrodes. Fig. 13 displays the variations in specific capacity and reversibility exhibited by a sample (*L* = 30 µm, ℓ = 10 nm) over 783 cycles at 0.5C (391 mA h g^−1^). Its behavior is representative of all samples presented above. As expected, the irreversible reactions leading to non-unity coulombic efficiency are completed within the first 30 cycles or so, after which the coulombic efficiency values are essentially 100% and the charge storage fully reversible. The capacity also drops by about 51% over the first 70 cycles, after which it not only stabilizes but even increases very slowly again. In fact, it almost reverts back to its original capacity (664 mA h g^−1^ of 724 mA h g^−1^). This unusual behavior is not specific to one particular sample or geometry but is observed in a qualitatively similar manner for all systems investigated. Similar behavior has been observed before in SnO_2_-based systems, without explanation being provided so far.^26,27,44^ Our hypothesis concerning the origin of this phenomenon is the following. In the initial stages of the material's lifetime, the initially continuous SnO_2_ layer is converted to a heterogeneous Sn/Li_2_O mixture in which the electrically conductive phase (Sn) may be holey, or even patchy. This increases ohmic losses and may even isolate some fraction of the electroactive material. Upon the effective kneading that occurs during subsequent charge/discharge cycles (with their associated volume changes), individual Sn particles or islands may well reconnect, thereby improving the electrical conductivity and the completeness of the conductive network. This scenario is consistent with the behavior observed for *Q*_dl_ over the course of 20 cycles.

## Conclusions

Taken together, the data presented establish the applicability of ALD in combination with ‘anodic’ porous substrates for the preparation of battery electrode materials in parallel nanotubular shape. Such arrays of parallel SnO_2_ nanotubes are active as a negative LIB electrode material in the absence of any additive. The nanotube structure is designed to have sufficient space for volume expansion during lithiation and shrinking upon delithiation and enables the experimentalist to explore systematically the geometric effects on performance and longevity. In this respect, the results are most convincing for thinner tube walls. The stabilized specific gravimetric capacity is highest (on the basis of SnO_2_) for tubes with 10 nm wall thickness. Not only does the presence of a void central space allow for volume changes without deleterious cracking and pulverization, it also seems to promote a slow recovery of electroactive material particles that may have lost contact with the bulk in the early stages of electrochemical cycling.

Our best samples compare positively with the state of the art for SnO_2_-based electrodes (Table 1), with a coulombic efficiency > 99% and a specific capacity of 671 mA h g^−1^ after 783 cycles. This represents a 92% retention of the initial capacity, and 86% of the theoretical value on the basis of SnO_2_. Since the best results on this scale are obtained for the samples that feature the thinnest walls, the areal specific capacity is low, as well as the overall gravimetric one (including the Al_2_O_3_ matrix). In other words, the highly tunable nature of this system allows the user to choose for each application the optimal geometry with either maximized longevity or maximized areal specific capacity, or a balance of both. In principle, the alumina matrix could even be removed after ALD generation of the electroactive material: this would cause a loss of order (since the tubes would tend to collapse), but would equate the gravimetric SnO_2_-only capacity (and energy density) with the overall gravimetric capacity of the negative electrode. These properties pave the way towards real-world applications of SnO_2_ in the lithium ion batteries of portable devices.

## Conflicts of interest

There are no conflicts to declare.

## Supplementary Material

NA-002-C9NA00799G-s001
