# Supplementary material for: Ordered SnO2 nanotube arrays of tuneable geometry as a lithium ion battery material with high longevity
Source: Nanoscale Adv. 2020 Feb 13;2(4):1417–26. doi: 10.1039/c9na00799g (PMC9417633; doi:10.1039/c9na00799g)
Supplement: NA-002-C9NA00799G-s001 [file NA-002-C9NA00799G-s001.pdf]

Electronic Supplementary Material (ESI) for Nanoscale Advances.
This journal is © The Royal Society of Chemistry 2020

Electronic Supplementary Information to

Ordered SnO_2 Nanotube Arrays of Tuneable Geometry as a Lithium Ion Battery Material with High Longevity

Ying Zhuo,[a] Sarah Tymek,[a] Hong Sun,[a] Maïssa K. S. Barr,[a,b] Lionel Santinacci,[b] and Julien Bachmann[a,c,*]

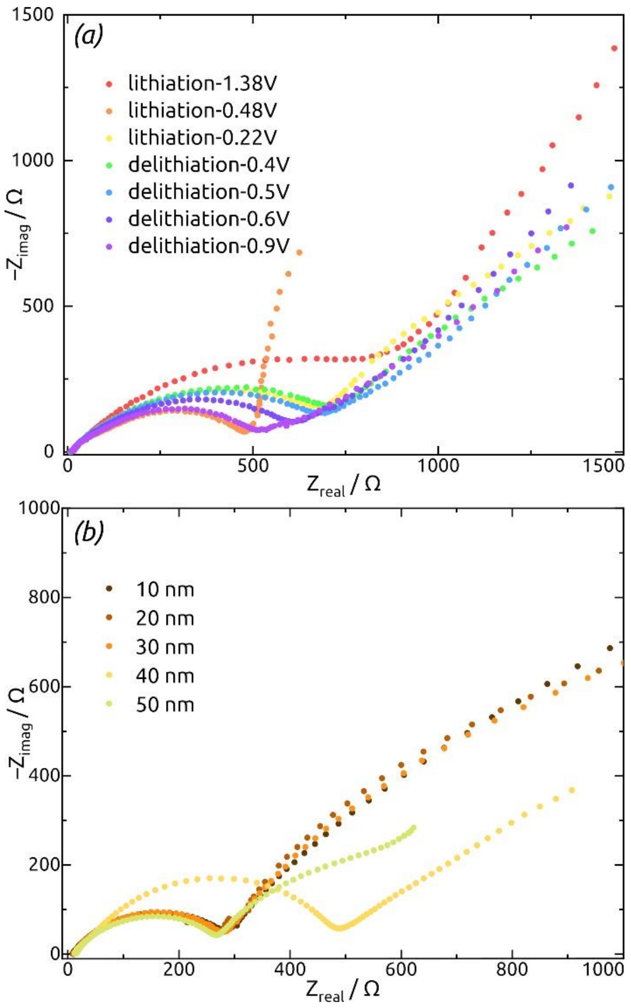

Figure S1. Nyquist plots of galvanostatic electrochemical impedance spectroscopy (EIS) measurements recorded of SnO_2 nanotube electrodes. Equivalent electrical circuit models shown in Figure 8 are used to fit the spectra of sample. *(a)* EIS measurements were performed on SnO_2 nanotube electrode (ℓ = 40 nm, L = 30 µm, 4 mm macroscopic sample diameter), which was charged/discharged to a certain voltage before EIS measurements. *(b)* EIS spectra for SnO_2 nanotube electrodes with different wall thicknesses in the delithiated state after 10 lithiation/delithiation cycles.

Table S1. Summary of EIS parameters fitted for various SnO_2-based systems. The first two datasets refer to a sample with 40 nm tube wall thickness at various stages of charge and discharge. The third dataset compares samples with various tube wall thicknesses in the discharged state at 2.80 V.

	R_s / Ω	Q_{SEI} / μS $s^{0.8}$	R_{SEI} / Ω	Q_{dl} / μS $s^{0.9}$	R_{sf+ct} / Ω
**lithiation**					
1.38 V	12.4 ± 0.2	42.5 ± 1.2	70.5 ± 11	5.9 ± 0.3	557.9 ± 10
0.48 V	9.4 ± 0.3	23.9 ± 0.6	116.1 ± 13	8.1 ± 0.7	286.8 ± 12
0.22 V	8.8 ± 0.2	25.5 ± 0.7	213.9 ± 45	8.1 ± 1.2	411.1 ± 39
**delithiation**					
0.40 V	9.4 ± 0.2	25.9 ± 0.8	237.3 ± 55	8.5 ± 1.4	426.3 ± 47
0.50 V	9.1 ± 0.2	25.3 ± 0.7	242.2 ± 52	9.1 ± 1.4	399.4 ± 46
0.60 V	10.2 ± 0.2	26.2 ± 0.7	210.5 ± 44	9.1 ± 1.4	356.4 ± 39
0.90 V	8.7 ± 0.2	23.7 ± 0.4	170.2 ± 20	10.0 ± 1.0	317.3 ± 18
**different thicknesses**					
10 nm	9.0 ± 0.1	3.7 ± 0.09	34.0 ± 2	2.3 ± 0.07	193.9 ± 2
20 nm	11.9 ± 0.1	8.0 ± 0.30	17.8 ± 1	3.4 ± 0.09	192.9 ± 2
30 nm	11.8 ± 0.1	3.7 ± 0.10	31.6 ± 3	1.8 ± 0.07	194.7 ± 3
40 nm	12.1 ± 0.1	2.4 ± 0.05	46.5 ± 2	1.8 ± 0.04	405.4 ± 3
50 nm	15.3 ± 0.2	3.0 ± 0.06	79.2 ± 5	3.4 ± 0.2	169.7 ± 5

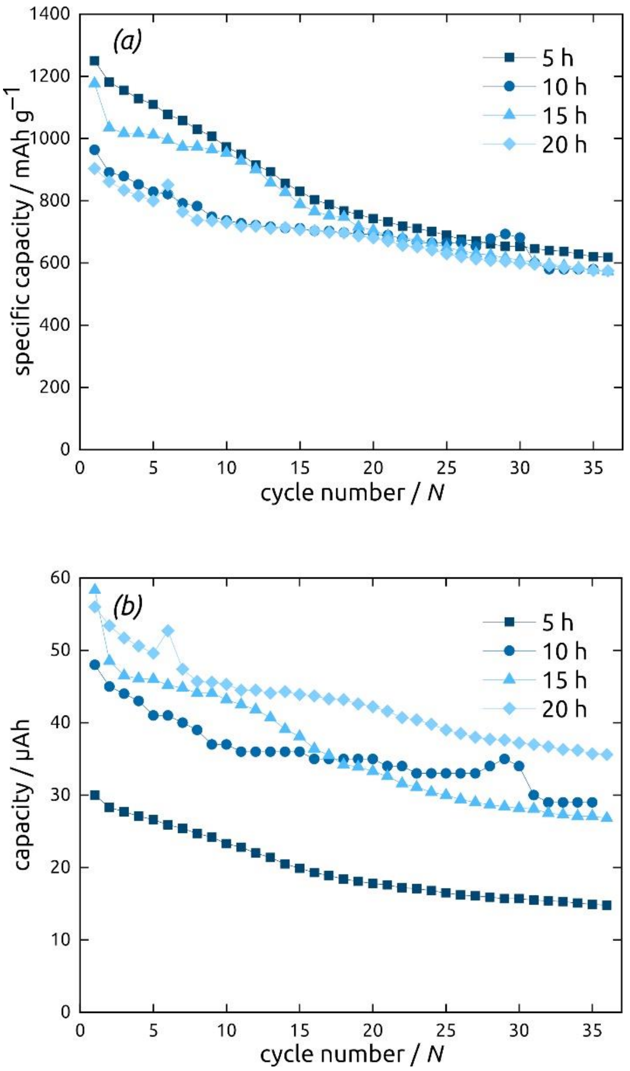

Figure S2. Galvanostatic charging and discharging curves of SnO_2 nanotube electrodes with 10 nm wall thickness. The length of SnO_2 nanotubes has been varied. *(a)* and *(b)* show specific capacity and absolute capacity of SnO_2 nanotubes with different lengths, respectively.
